# STEMI Outcomes in Guangzhou and Hong Kong: Two-Centre Retrospective Interregional Study

**DOI:** 10.1371/journal.pone.0149981

**Published:** 2016-03-09

**Authors:** Xiaohui Chen, Min Li, Huilin Jiang, Yunmei Li, Junrong Mo, Peiyi Lin, Colin A. Graham, Timothy H. Rainer

**Affiliations:** 1 Emergency Department, The 2nd Affiliated Hospital of Guangzhou Medical University, Guangzhou, China; 2 Accident and Emergency Medicine Academic Unit, Chinese University of Hong Kong, Hong Kong, China; Innsbruck Medical University, AUSTRIA

## Abstract

**Background and Objectives:**

Healthcare systems are organized very differently in Hong Kong (HK) and Guangzhou (GZ). This study compared managements of the emergency departments (ED) and one-year mortalities of ST-segment elevation myocardial infarction (STEMI) patients in two teaching hospitals in Guangzhou and Hong Kong.

**Methods:**

Retrospective observational study of STEMI mortalities and treatments in the Prince of Wales Hospital (PWH) and the Second Affiliated Hospital of Guangzhou Medical University (AHGZMU), was conducted between January and December 2010. The primary outcome was one-year all cause mortality.

**Results:**

Univariate analysis of 76 cases from PWH and 111 cases from AHGZMU showed similar clinical characteristics, except for lower proportions of males (74% vs 92%, P = 0.002), hyperlipidemia (5% vs 25%, P<0.001), and Killip class I (56% vs 91%; P<0.001) in AHGZMU. The onset-to-door time of STEMI patients in AHGZMU was longer than in PWH (median 205 min [(IQR: 95–432) vs 120 min (IQR: 55–225), P = 0.001]. In AHGZMU, 85 (77%) patients received primary percutaneous coronary intervention (PPCI) as the main reperfusion treatment, whereas 18 (24%) received PPCI and 51 (67%) patients received thrombolytic therapy in PWH. Overall the one-year mortality in AHGZMU was 20%, whilst in PWH it was 14% (P = 0.436). The standardized one-year all-cause mortality ratios for AHGZMU and PWH were comparable (18.7 vs. 18.2%, P = 0894). Independent predictors of one-year mortality included older age (>67 years) and hyperglycemia (>10 mmol/L). Aged over 65 years, presence of anterior wall infarct, body weight ≤65 kg, SBP <100 mmHg at ED and glucose level >10 mmol/L were the independent predictors of in-hospital MACE.

**Conclusion:**

There was no statistically significant difference between the standardized one-year all-cause mortalities of STEMI patients in the setting mainly using thrombolysis with shorter door-to-treatment time and the setting mainly using PCI with longer door-to-treatment time. Aged over 67 years and glucose level over 10 mmol/L were the independent predictors of one-year mortality. Older age, presence of anterior wall infarct, lower body weight, lower SBP at ED and hyperglycemia were the independent predictors of in-hospital MACE.

## Introduction

The World Health Organization reported that coronary artery disease (CAD) is the commonest cause of death worldwide, claiming 17 million deaths in 2008[1]. In US, coronary heart disease is the most common type of heart disease, killing more than 385,000 people annually [2]. In Hong Kong, heart disease is the second commonest cause of death, claiming 4,361 lives in 2011, with coronary heart disease accounting for 42.2% of total cardiac mortality [3]. Sino-MONICA (Monitoring Trends and Determinants in Cardiovascular Disease) study has reported that the CHD mortality increased by 41% in men and 39% in women for the age group 35–74 years [4,5].

In order to improve the quality of survival of cardiac patients, there is a worldwide impetus to develop and improve cardiac systems[6]. Both Hong Kong and Guangzhou have advanced systems of cardiac care[7,8]. Organizational and wider system factors have been consistently shown to have a significant influence on organizational change, success or failure of implementation of interventions, and their effectiveness [9].

It is important to look beyond mere survival, and to assess the reasons for differences in survival. Patient-centred, health-related outcomes are increasingly recognized as an important benchmark of the quality of care received. Meaningful comparisons between different centres enable healthcare providers to assess how well they are doing and where they might target future development. Comparable registries have been developed in Guangzhou and Hong Kong.

There is little information about the recovery of patients following ST elevation myocardial infarction (STEMI) in Hong Kong and Guangzhou. The aim of this retrospective observational study was to compare post-STEMI one-year all-cause mortalities and in hospital major adverse cardiac events (MACE) in patients treated in the Prince of Wales Hospital (PWH) in Hong Kong and the Second Affiliated Hospital of Guangzhou Medical University (AHGZMU) in Guangzhou, both in Southern China. We also investigated any significant differences between clinical practices in PWH and AHGZMU, and whether these might contribute to mortality or MACE outcomes.

## Methods

### Study design

Ethical approval was obtained from the joint Chinese University of Hong Kong-New Territories East Cluster Clinical Research Ethics Committee in Hong Kong and the Institutional Review Board in Guangzhou to conduct a retrospective study in patients with STEMI. All patient records/information was anonymized and de-identified prior to analysis. The period of study, including recruitment and follow up, was from 1 January 2010 to 31 December 2010.

### Study Setting

AHGZMU serves a population of approximately 1.56 million people in the Hai Zhu district, Guangzhou. AHGZMU is an academic hospital with 1500 beds affiliated with the Guangzhou Medical University. The emergency department (ED) receives more than 150 000 new patients per annum and serves a local population of approximately 1550 000 people.

In Hong Kong, the population is about 7 million of which 95% are Chinese. PWH is located in the New Territories in Hong Kong; it is a university hospital with 1400 beds. It sees more than 150 000 new ED patients per annum and serves a local population of approximately 800 000 people.

System-level barriers affect the ability of acute myocardial infarction (AMI) clinical pathways to change practice. The healthcare systems of the two cities are quite different under the “one country, two systems” policy. Mainland China has integrated many features of health care systems associated with market economies, while its overall economy is largely centrally planned. In contrast, Hong Kong has adopted health care financing and organizational health systems that are commonly seen in centrally planned economies, while its economy functions as a highly capitalistic enterprise [10]. The hospitals in Hong Kong adopt the ED management system are come from England. By comparing the ED management systems in both cities may allow us to find the influence of these system barriers on the healthcare quality.

In both settings, patients have to pay for healthcare services. However, it is more expensive in Guangzhou than in Hong Kong relative to the living cost. On the other hand, the principal modes of reperfusion in Guangzhou and in PWH are primary PCI and thrombolysis respectively.

### Inclusion and exclusion criteria

All adult patients aged ≥18 years with STEMI at ED presentation between 1 January 2010 and 31 December 2010 were included. Patients were excluded if they had STEMI with onset >24h, if they had NSTEMI, or if they were transferred from other hospitals.

### Measurements and Data Collection

Patient characteristics, including age, sex, infarct location, cardiac risk factors, initial observations in ED, in-hospital comorbidities, electrocardiographic changes, Killip classification, renal function, troponin levels, glucose, and time from ED arrival to administration of thrombolysis therapy (‘door-to-needle’), or time from ED arrival to inflation of the angioplasty balloon at PPCI (‘door-to-balloon’) were collected. The one-year all-cause mortality) and in-hospital MACE, including in-hospital mortality, reinfarction and cardiogenic shock, were recorded. All deaths and admission records in the Hong Kong public health care system are recorded on the territory-wide clinical management system (CMS). Patients in Hong Kong were followed up for one year by reviewing CMS records. Patients in Guangzhou were followed up for one year by reviewing the medical records whilst the one-year mortality was checked by reviewing the Public Security System records.

### Definitions

Acute coronary syndromes (ACS) is an umbrella term for a spectrum of symptoms that are compatible with acute myocardial ischaemia [11], consisting of unstable angina, Non ST-segment elevation myocardial infarction (NSTEMI) and STEMI [12]. STEMI is an ECG appearance of ST segment elevation of ≥0.1mV) in two contiguous limb leads, or ST segment elevation of >0.2mV in two or more contiguous chest leads; or new or presumed new left bundle branch block. STEMI should be associated with symptoms consistent with ACS and abnormally elevated cardiac troponin levels.

PPCI was defined as all primary angioplasty procedures with or without stenting within 12 h for STEMI patients. PPCI is to open in the infarct-related (‘culprit’) coronary artery and restoring coronary blood flow as quickly as possible [13].

Onset to door time (prehospital delay) was defined as the onset of symptoms to the registration time in the ED.

Door to ECG time was defined as the time from registration to ECG test.

Door -to-needle time was defined as the time from ED arrival to thrombolysis.

Door-to-balloon time was defined as the time from ED arrival to PPCI Time.

In-hospital major adverse cardiac events in hospital (MACE) were defined as those relating to safety outcomes, including all-cause mortality (including cardiac death and sudden cardiac death), readmission with myocardial infarction and cardiogenic shock. One-year all-cause mortality was defined as the percentage of patients who died from all causes within one year after ED presentation.

### Sample size calculation

According to the study reported by Duan et al [14], the one-year mortality rates in STEMI patients with thrombolytic treatment was 37.5% and with PCI treatment was 15.1% before establishment of the regional collaborative network. To achieve adequate power to address the objectives by using 2-tailed alpha of 0.05 and a power of 80%, the minimum sample size required per group was 57. We aimed to recruit an extra 30% in case for unforeseen circumstances and thus at least 74 (57 x 1.3 = 74) patients were required per group. Therefore, the minimum sample size in this study was 148.

### Statistical analyses

Summary statistics were used to describe patient characteristics from the Hong Kong and Guangzhou groups. Chi-square analysis was used for categorical variables, whilst independent t-tests or Mann-Whitney U-tests were used for comparing data from continuous variables.

An initial univariate analysis was performed on all variables with one-year mortality and in-hospital MACE as the dependent variables and presented as unadjusted odds ratios (OR) and 95% confidence intervals (95% CI). Then variables with p value <0.05 were entered into a multivariate ordinal logistic regression and presented as adjusted ORs and 95% CI. Statistical significance was set at p<0.05. All analyses were performed using SPSS v17.0 (SPSS Inc, IL, USA) and Medcalc v9.5 (MedCalc Software, Mariakerke, Belgium).

## Results

### Recruitment of patients from the two centres

**Fig 1:**
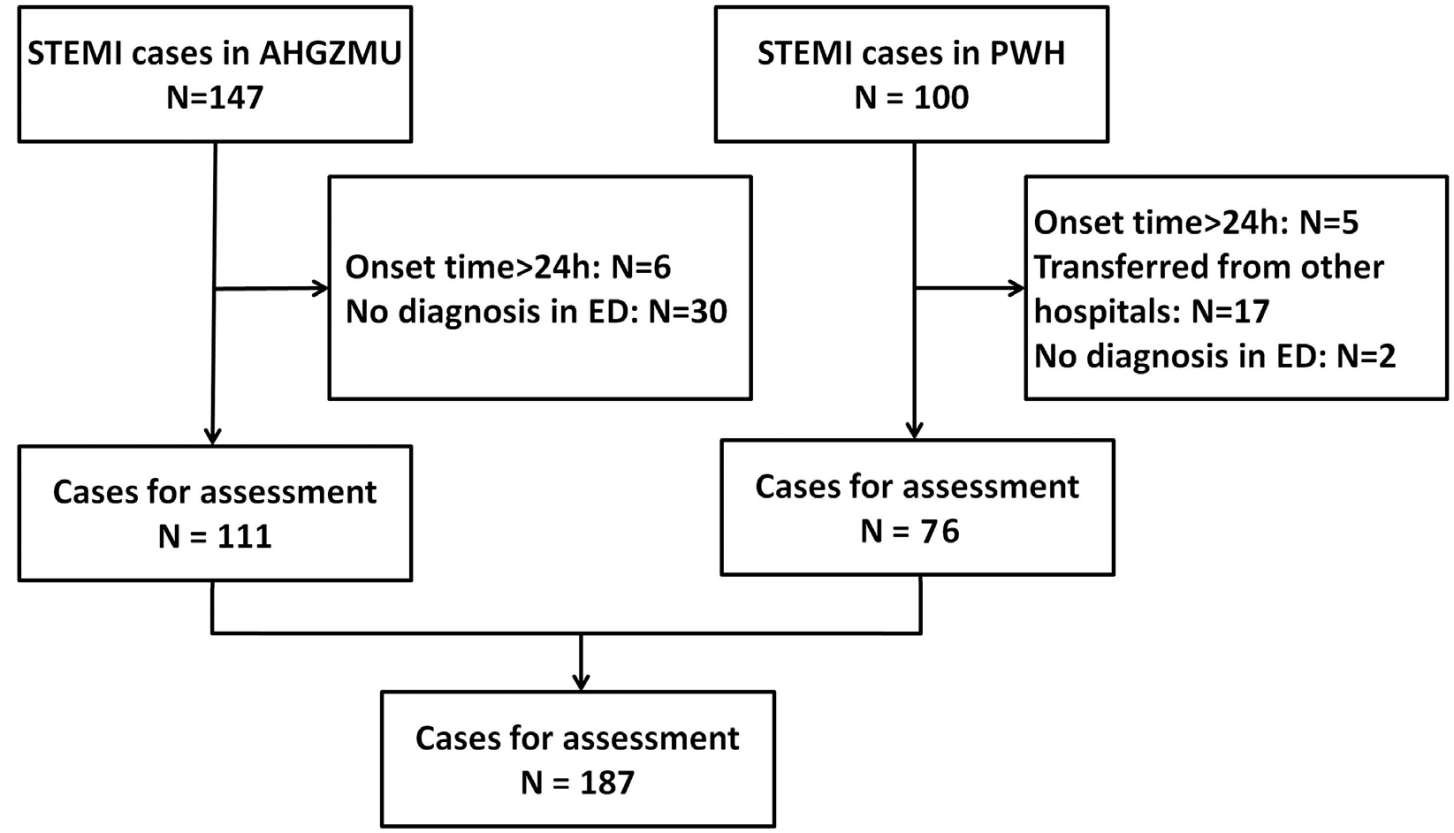
Flow chart of STEMI patient recruitment in two hospitals.

Between 1 January 2010 and 31 December 2010, 247 patients with STEMI were enrolled in our study (Fig 1). 60 patients were excluded due to 11 cases with onset time>24h, 17 cases transferred from other hospitals and 32 cases without diagnosis in ED, leaving 187 patients (111 cases from AHGZMU and 76 cases from PWH) for inclusion to the study.

### Study population characteristics

Table 1 shows the characteristics of STEMI patients from AHGZMU and PWH. Compared to patients in PWH, patients in AHGZMU had lower proportions of male and hyperlipidaemia, and Killip class I, lower blood pressure, and longer symptom onset to ED arrival time. Also, patients in AHGZMU had shorter ED arrivals to ECG times, and shorter door-to-balloon times. In AHGZMU, 77% of patients received primary PCI. In PWH, 24% of patients received primary PCI, whilst 57% had thrombolytic therapy. There were no significant differences in one year all-cause mortality or in-hospital MACE between AHGZMU and PWH. Overall the one-year mortality in AHGZMU was 20%, whilst in PWH it was 14% in PWH (P = 0.436).

**Table 1:** Comparison of patient characteristics between the two hospitals (N = 187).

	AHGZMU (n = 111)	PWH (n = 76)	*P Value*
Age, years, mean (SD)	68 (14)	64 (13)	0.059
Males, n (%)	82 (74)	70 (92)	0.002*
Infarct locations			
Anterior wall, n (%)	61 (55)	40 (53)	0.754
Non-anterior wall, n (%)	50 (45)	36 (47)	
**Risk factors and in-hospital comorbidities**			
Smoker, n (%)	44(40)	29(38)	0.838
Hypertension, n (%)	56(50)	39(51)	0.812
Coronary artery disease, n (%)	22(20)	16(21)	0.837
Diabetes, n (%)	19(17)	16(21)	0.498
Hyperlipidemia, n (%)	6(5)	19(25)	<0.001*
Renal inadequacy, n (%)	2(1.8)	4(5.3)	0.191
Cancer, n (%)	1(1.0)	3(3.9)	0.161
Infection (pneumonia, urinary tract infection), n (%)	30(27.0)	4(5.3)	<0.001*
Hyperuricemia, n (%)	2(1.8)	1(1.3)	0.789
COPD, n (%)	2(1.8)	2(2.6)	0.707
Arterial thrombosis, n (%)	2(1.8)	1(1.3)	0.789
Aneurysm, n(%)	0	2(2.6)	_
**First ED characteristics**			
Heart rate, bpm, mean (SD)	79 (27)	73 (19)	0.082
Systolic blood pressure, mmHg, mean (SD)	123 (34)	137 (34)	0.008*
Diastolic blood pressure, mmHg, mean (SD)	76 (21)	77 (22)	0.704
Killip classification:			
Killip class I n (%)	62 (56)	69 (91)	<0.001*
Killip classII n (%)	24 (22)	0 (0)	
Killip class III n (%)	11 (10)	6 (8)	
Killip class IV n (%)	14 (13)	1 (1)	
**First lab results in ED:**			
Creatinine, μmoL/L, median (IQR)	103(90–123)	98.5(81–126)	0.092
Glucose, mmoL/L, mean (SD)	9.24 (5.09)	8.99 (4.14)	0.719
**Illness onset to ED time**			
Onset-to- door time, min, median (IQR)	205(95–432)	120(55–225)	0.001*
Distribution of onset-door time			
<3 hours, n (%)	48 (43)	50 (66)	<0.001*
3 to 6 hour, n (%)	26 (23)	17 (22)	
≥6 hours, n (%)	37 (33)	6 (8)	
**Procedures and Times**			
ED arrival-ECG time, min, median (IQR)	5 (3–6)	9 (5–14)	<0.001*
Received reperfusion therapy, n (%)	85(77)	69 (91)	0.012*
Thrombolytic therapy, n (%)	0 (0)	51 (67)	
Primary PCI, n (%)	85(77)	18(24)	
ED arrival-to-reperfusion time			
Overall door-to treatment time, min, median (IQR)	150(80–612.5)	39(25–95)	<0.001*
Door-to-needle time, min, median (IQR)	0 (0)	31 (21–49)	
Door-to-balloon time, median (IQR)min	150(80–612.5)	182(85–4843)	0.003*
**Performance Targets**			
Overall number of patients treated within the recommended reperfusion time, n (%)	29 (26)	29 (38)	0.081
Number of patients treated within the recommended ED door-to-needle time ≤30min, n (%)	0	25(33)	<0.001*
Number of patients treated within the recommended ED door-to-balloon time ≤90min, n (%)	29 (26)	4 (6)	<0.001*
**Outcomes**			
One-year all-cause mortality, n (%)	22 (20)	11 (14)	0.436
In hospital MACE, n (%)	23 (21)	16 (21)	0.956
Mortality alone, n (%)	16 (14)	9 (12)	0.672
Reinfarction alone, n (%)	2 (2)	5 (7)	0.122
Cardiogenic shock alone, n(%)	15 (14)	14 (18)	0.413
Hospital length of stay, mean (SD), days	10(6)	6(3)	<0.001*

*statistically significant

Twenty-six patients in AHGZMU and 7 patients in PWH did not receive reperfusion therapy. In AHGZMU, 16 patients (62%) refused reperfusion therapy, 8 patients (31%) were in unstable conditions (including cardiogenic shock and Malignant arrhythmias), and the reasons of another 2 patients not to receive reperfusion therapy were unknown. In PWH, 2 patients (29%) were delayed at presentation, 2 patients (29%) were in unstable conditions, 1 patient (14%) had typical ECG at ED admission, and 2 patients had unknown reasons not to receive reperfusion therapy.

Sixteen patients in AHGZMU and 9 patients in PWH died during hospital stay. The causes of in-hospital mortality were cardiogenic shock (9 cases in AHGZMU and 6 cases in PWH), fatal arrhythmias (5 cases in AHGZMU and 3 cases in PWH) and non-cardiogenic reasons (2 cases in AHGZMU, including one died from cerebral infarction and another one died from acute mesenteric artery embolism, and 0 case in PWH).

### Unadjusted and adjusted odd ratios for one-year all-cause mortality

Table 2 shows that patients were more likely to die if they were older, had anterior wall infarcts, had lower body weights, histories of hypertension, lower SBP and DBP at ED admission, worse Killip class and higher blood glucose level. After adjusted for age, presence of anterior wall infarct, body weight, past history of hypertension, Killip class and blood glucose, the adjusted odds of death in PWH compared with AHGZMU was 0.685 (95% CI:0.310–1.510). Independent predictors of one-year mortality included older age (>67 years) and hyperglycemia (>10 mmol/L).

**Table 2:** Unadjusted and adjusted odds ratios for one-year all-cause mortality (N = 187).

	Unadjusted Odds Ratio (95% CI)	*P*	Adjusted Odds Ratio* (95% CI)	*P*
Hospital				
PWH	0.685(0.310–1.510)	0.348		
AHGZMU	Reference			
Age (years)	1.081(1.043–1.122)	<0.0001	1.085(1.03–1.15)	0.005*
Gender				
Female	0.542(0.226–1.299)	0.169		
Male	Reference			
Infarct locations			1.885(0.589–6.033)	0.286
Non-anterior wall	0.446 (0.199–1.000)	0.050		
Anterior wall	Reference			
Weight (kg)	0.935(0.880–0.993)	0.028	0.943(0.875–1.017)	0.943
**Risk factors**				
CAD				
Yes	1.957(0.838–4.568)	0.121		
No	Reference			
Hypertension				
Yes	3.354(1.461–7.699)	0.004	0.560(0.181–1.738)	0.683
No	Reference			
Stroke				
Yes	2.205 (0.705–6.895)	0.174		
No	Reference			
Diabetes				
Yes	2.229 (0.942–5.274)	0.068		
No	Reference			
Hyperlipidemia				
Yes	1.579 (0.442–5.639)	0.567		
No	Reference			
Smoker				
Yes	1.379 (0.646–2.946)	0.413		
No	Reference			
**Onset-ED time period:**				
<3h	1.080 (0.408–2.855)	0.877		
≥3h-6h	0.949 (0.305–2.948)	0.857		
≥6h	Reference			
ED arrival to reperfusion time within recommended time				
Yes	0.661 (0.280–1.580)	0.356		
No	reference			
**Killip classification**			1.232(0.747–2.031)	0.414
Killip class IV	4.818(1.405–16.521)	0.008		
Killip class III	3.929(1.263–12.224)	0.017		
Killip class II	1.903(0.565–6.417)	0.264		
Killip class I	Reference			
**Laboratory Investigations**				
Troponin (ng/L)	0.586 (0.112–2.382)	0.39		
Glucose (mmol/L)	1.145(1.062–1.235)	<0.0001	1.187(1.047–1.346)	0.008*

* All variables with P<0.05 were entered into the model. The model was adjusted for age, anterior wall infarct, weight, risk factors of hypertension, Killip classification and blood glucose level.

### Unadjusted and adjusted odds ratios for in-hospital MACE

The unadjusted odds ratios in PWH (AHGZMU as reference) for in-hospital mortality alone was 0.798 (95%CI 0.333–1.912, p = 0.612), for in-hospital reinfarction alone was 3.838 (0.725–20.334; p = 0.114), and for cardiogenic shock alone was 1.445 (0.652–3.021; p = 0.364). After adjusting for age, anterior wall infarct, weight, past history of hypertension, Killip class and blood glucose, the independent predictors of in-hospital MACE included older age (>65 years), presence of anterior wall infarct, lower body weight (≤65 kg), lower SBP (<100 mmHg) at ED and hyperglycemia (>10 mmol/L) (Table 3).

**Table 3:** Unadjusted and adjusted odds ratios for in-hospital MACE (N = 187).

	Unadjusted Odds Ratio (95% CI)	*P*	Adjusted Odds Ratio* (95% CI)	*P*
Hospital				
PWH	1.020 (0.489–2.091)	0.956		
AHGZMU	Reference			
Age (years)	1.053 (1.022–1.084)	<0.001	1.069 (1.026–1.129)	0.003*
Gender				
Female	0.493 (0.216–1.122)	0.092		
Male	Reference			
Infarct locations				
Non-anterior wall	0.383 (0.177–0.824)	0.014	0.262 (0.077–0.921)	0.035*
Anterior wall	Reference			
Weight (kg)	0.893 (0.837–0.953)	0.001	0.918 (0.84 2–0.990)	0.027*
**Risk factors:**				
CAD				
Yes	1.761 (0.781–3.968)	0.173		
No	Reference			
Hypertension				
Yes	1.256(0.624–2.567)	0.514		
No	Reference			
Stroke				
Yes	1.832 (0.596–5.624)	0.290		
No	Reference			
Diabetes				
Yes	1.114 (0.450–2.717)	0.817		
No	Reference			
Hyperlipidemia				
Yes	1.367 (0.493–4.261)	0.590		
No	Reference			
Smoker				
Yes	1.449 (0.711–2.955)	0.307		
No	Reference			
Systolic BP (mmHg)	0.984 (0.974–0.994)	0.003	0.969 (0.937–0.991)	0.046*
Diastolic BP (mmHg)	0.981 (0.965–0.997)	0.023	1.016 (0.956–1.069)	0.503
Heart rate (bpm)	1.005 (0.991–1.020)	0.458		
Onset-ED time period:				
<3h	0.758 (0.294–1.955)	0.567		
≥3h-6h	1.343 (0.577–3.115)	0.496		
≥6h	Reference			
ED arrival to reperfusion time within recommended time				
Yes	1.526 (0.731–3.187)	0.260		
No	reference			
**Killip classification**				
Killip class IV	5.66 2(1.860–17.234)	0.002		
Killip class III	2.702 (0.904–8.079)	0.074		
Killip class II	0.708 (0.194–2.580)	0.601		
Killip class I	Reference			
Glucose (mmol/L)	1.126 (1.047–1.210)	0.001	1.153 (1.013–1.431)	0.037*

*All variables with P<0.05 were entered into the model. The model was adjusted for age, anterior wall infarct, weight, systolic and diastolic blood pressure, Killip classification and blood glucose level.

### Standardized one-year all-cause and in-hospital mortality ratios of STEMI patients

Table 4 shows that the higher Killip class was associated with the higher one-year all-cause and in-hospital mortalities in both hospitals. The standardized one-year all-cause mortality ratios for AHGZMU and PWH were comparable (18.7% vs. 18.2%, P = 0.894). The standardized in-hospital mortality ratio of PWH was slightly higher than that of AHGZMU (16.6% vs. 14.4%, P = 0.568).

**Table 4:** Standardized one-year all-cause and in-hospital mortality ratios of STEMI patients.

	Overall population	AHGZMU			PWH		
		Actual mortality		Expected number of deaths	Actual mortality		Expected number of deaths
**One-year all-cause mortality**							
Killip class I	131	10/62 (16.1%)		21	6/69 (8.7%)		11
Killip class II	24	5/24 (20.8%)		5	0 (0%)		0
Killip class III-IV	32	7/25 (28.0%)		9	5/7 (71.4%)		23
Overall	187	22/111 (19.8%)		35	11/76 (14.5%)		34
*Standardized mortality ratio		35/187 (18.7%)			34/187 (18.2%)		
**In-hospital mortality**							
Killip class I	131	8/62 (12.9%)		17	4/69 (5.8%)		8
Killip class II	24	2/24 (8.3%)		2	0 (0%)		0
Killip class III-IV	32	6/25 (24.0%)		8	5/7 (71.4%)		23
Overall	187	16/111 (14.4%)		27	9/76 (11.8%)		31
^#^Standardized mortality ratio		27/187 (14.4%)			31/187 (16.6%)		

*Comparison of the standardized mortality ratios of the two hospitals (P = 0.894)

^#^Comparison of the standardized mortality ratios of the two hospitals (P = 0.568)

### Comparison of in-hospital and one-year mortalities in STEMI patients with and without reperfusion therapy

Table 5 shows the comparison of in-hospital and one year mortalities in STEMI patients with reperfusion and without reperfusion therapy. The majority of patients received reperfusion. The in-hospital and one year mortalities in STEMI patients receiving reperfusion were lower than those patients without reperfusion. There was no significant difference in in-hospital and one year mortalities between the two hospitals.

**Table 5:** The Comparison of in-hospital and one-year all-cause mortality in STEMI patients with and without reperfusion therapy.

	With Received Reperfusion				Without Received reperfusion			
	AHGZMUn = 85	PWH n = 69	Total N = 154	P value	AHGZMU n = 26	PWH n = 7	Total N = 33	P value
In-hospital mortality, n (%)	8 (9)	6 (10)	14 (9)	0.878	8 (30)	3 (42)	11 (33)*	0.390
One year mortality, n (%)	14 (16)	8 (12)	22 (14)	0.547	8 (31)	3 (42)	11 (33)*	0.390

*Compared with the total number of patients received reperfusion (P<0.05)

### Comparison of characteristic of STEMI patients in Killips class I and Killips class ≥II

Table 6 shows that patients in Killip class ≥II were older, had higher proportion of females patients, lower proportions of hyperlipidemia and receipt of reperfusion therapy, higher blood glucose and worse outcomes.

**Table 6:** Comparison of characteristics of STEMI patients in Killip class I and Killip class ≥II.

	Killip class 1 (n = 131)	Killip class ≥II (n = 56)	P Value
Age, years, mean (SD)	64 (14)	72 (13)	<0.001*
Males, n (%)	116(89)	36 (64)	<0.001*
Infarct locations			
Anterior wall, n (%)	68 (52)	33 (59)	0.378
Non-anterior wall, n (%)	63 (48)	23 (41)	
Risk factors			
Hypertension, n (%)	61 (47)	32 (57)	0.185
Coronary artery disease, n (%)	27 (21)	17 (20)	0.880
Diabetes, n (%)	25 (20)	11 (20)	0.989
Hyperlipidemia, n (%)	23 (18)	2 (4)	0.010
First ED characteristics:			
Heart rate, bpm, mean (SD)	75 (22)	81 (29)	0.093
Systolic blood pressure, mmHg, mean (SD)	131 (32)	121 (39)	0.075
Diastolic blood pressure, mmHg, mean (SD)	77 (20)	73 (24)	0.275
First lab results in ED:			
Glucose, mmoL/L, mean (SD)	8.50 (3.53)	10.64 (6.51)	0.040*
Treatment			
Received reperfusion therapy, n (%)	114 (87)	40(71)	0.001*
Outcomes			
One-year all-cause mortality, n (%)	16 (12)	17 (30)	0.003*
In hospital MACE, n (%)	22 (17)	17 (30)	0.037*

*statistically significant.

## Discussion

The standardized one-year all-cause mortality ratios for STEMI patients in Hong Kong and Guangzhou were similar despite the differences in acute management between the two cities. This is the first study to compare the mortality and MACE rate of STEMI patients in Hong Kong and Guangzhou, where healthcare systems are organized very differently. Different healthcare systems cause differences in ED management approaches to disease. These differences in management were clearly demonstrated in our study.

### Characteristics and ED management of STEMI patients in two hospitals

There were differences in main reperfusion approaches between Hong Kong and Guangzhou. In AHGZMU, the majority of patients received PPCI as the main reperfusion treatment, whereas in PWH only a small proportion received PPCI and two thirds of patients received thrombolytic therapy. Also, a lower proportion of STEMI patients received reperfusion therapy in AHGZMU (77% vs 91% in PWH).

We noted longer pre-hospital time delays and in-hospital delays in AHGZMU. Previous studies have shown that pre-hospital delay is a significant problem worldwide [15–17]. A multicentre registry in China found the median prehospital delay time for patients with AMI was 4 hours [18].

In-hospital delays in PWH were short and this was associated with the utilization of straightforward intravenous thrombolytic therapy as the main reperfusion treatment. The in-hospital delays to PPCI in AHGZMU (150 min) and in PWH (182min) were severe. Similar results have been found in a multicentre study in China which demonstrated the median in-hospital delay to primary angioplasty for patients with STEMI was 135 minutes [19]. Only 16.9% of the patients had door-to-balloon times of 90 minutes or less, and nearly a quarter of patients had the times in excess of 180 minutes [19].

Older age, lower systolic BP, lower body weight, anterior infarct location and higher blood glucose level were dependent factors for inhospital MACE. Older age and higher glucose were dependent factors for one-year mortality. Similar results were verified by the other clinical trials [20–23]. Prevention and control of risk factors is a key way to reduce in-hospital MACE and mortality [24–26].

### Factors accounting for discrepancies in treatment in the two hospitals

The dissimilarity of healthcare systems in the two cities is the main reason for the different ED management. In mainland China, limited capacity of hospital emergency care services, high out-of-pocket expenses with the need for up-front payment, prolonged discussions with the patient and families for both obtaining consent and pooling funds are likely to be major contributing factors for the long pre-hospital, in-hosptial delay and lower reperfusion rate[10,27].

Reperfusion therapy is the key treatment for STEMI, Our study showed that the in-hospital and one-year mortalities of STEMI patients with reperfusion were lower than those without reperfusion. Therefore, STEMI patients are encouraged to have reperfusion therapy to improve the mortalities and MACE [13].

Longer time to reperfusion and lower reperfusion rate in AHGZMU were associated with a higher proportion of patients in worse Killip class. The Killip classification is a simple clinical tool in cardiovascular assessment and risk stratification of patients with STEMI [18,28–29]. Patients with a higher Killip class have more severe angiographic coronary artery diseases, a higher incidence of ventricular dysfunction, and larger myocardial infarctions [18, 29]. Those patients in higher Killip classes with poorer conditions had less chance to receive reperfusion therapy and thus had worse outcomes. Expanding the health insurance coverage should be a priority of health reform to resolve these problems.

Using PPCI as a sole reperfusion therapy is another problem in AHGZMU management. Though PPCI is associated with normal epicardial flow in more than 90% of patients [30,31], PPCI is associated with significantly lower 30-day mortality relative to fibrinolysis, regardless of treatment delay. It is simply not feasible for all STEMI patients to receive PPCI [32]. Fear of disputes with patients and possible consequences of litigation and negative publicity for clinicians and hospitals has a major influence on thrombolysis treatment approach in Mainland China [9]. Therefore available fibrinolytic therapy should be adopted in AHGZMU to improve the reperfusion rate and shorten the in-hosptial delay in AHGZMU.

The overall adjusted one-year all-cause mortality and in-hospital MACE for STEMI patients in Hong Kong and Guangzhou were similar despite longer pre-hospital and in-hospital delay and lower reperfusion rate in AHGZMU. This may be explained by the thrombolytic therapy as the primary reperfusion approached in PWH. Lower PPCI rate in PWH was associated with unavailability of 24-hour on-call cardiac team [7]. Guidelines state that STEMI patients should be treated with thrombolytic therapy within 30 minutes [33]. The time from door-to-needle was still long in PWH. Although PWH had a higher reperfusion rate and shorter door-to-needle times, only 38% of patients were treated within the recommended reperfusion target time. The benefits of timely reperfusion treatment reinforce the importance of a comprehensive approach to cardiac care for all STEMI patients[32]. STEMI patients treated with fibrinolysis within 2 hours of symptom onset had a significantly lower 5-year mortality rate compared with those managed with PPCI[34]. However, normal coronary flow was restored in only 29–54% of patients receiving fibrinolysis compared with patients treated with PPCI [32,35]. Physicians always face decision-making dilemma on the optimal reperfusion therapy approach in the real world. These delays are detrimental to patients and can be exaggerated by variations in timing of patients' presentation and diagnosis. Optimizing the revascularisation strategies, increasing the PPCI rate and early revascularization patency would reduce the MACE rate and mortality in STEMI patients[36–37].

The current study has two limitations. Firstly, the data was collected from a retrospective observational study. There was no assessment study on the inter-rater and intra-rater reliability of the physicians' determinations. Secondly, The sample size is too small, However, this is the first study to compare the clinical outcomes of STEMI patients in Hong Kong and Guangzhou, where both cities are in China, but healthcare systems are under the “one country, two systems” policy. Also, this was a pilot study for preparation of a prospective multicentre study.

In conclusion, there was no statistically significant difference between the standardized one-year all-cause mortalities of STEMI patients in the setting mainly using thrombolysis with shorter door-to-treatment time and the setting mainly using PCI with longer door-to-treatment time. Age over 67 years and glucose level over 10 mmoL/L were the independent predictors of one-year mortality. Older age, presence of anterior wall infarct, lower body weight, lower SBP at ED and hyperglycemia were the independent predictors of in-hospital MACE. In the present study, we recognized the differences in STEMI patient management between the two hospitals and the key factors contributed to the one-year all-cause mortality and MACE of STEMI patients in Hong Kong and Guangzhou, and thus might allow us to further improve our clinical management systems for those patients.

## Abbreviations

ACS: Acute coronary syndrome
AHGZMU: The Second Affiliated Hospital of Guangzhou Medical University
CABG: Coronary artery bypass grafting
CAD: Coronary artery disease
CMS: Clinical Management System
cTnT: Cardiac troponin T
ED: Emergency department
IQR: Interquartile range
MACE: Major adverse cardiac events
NSTEMI: Non ST-segment elevation myocardial infarction
PPCI: Primary percutaneous coronary intervention
PWH: Prince of Wales Hospital
STEMI: ST elevation myocardial infarction
TIMI: Thrombolysis in myocardial infarction
US: United States
